# From Anatomy to Quality of Life: The Impact of Physiotherapy on Limb Changes in Cancer-Related Lymphedema

**DOI:** 10.7759/cureus.90872

**Published:** 2025-08-24

**Authors:** Irene Katsika, Evangelos Dimakakos, Margarita G Toumanidou, Alexandros Manthas, Dimitrios Nikas, Dimitrios Vergados, Pavlos Myrianthefs, Theodoros Mariolis-Sapsakos

**Affiliations:** 1 Department of Physiotherapy, General Hospital of Athens "G. Gennimatas", Athens, GRC; 2 Department of Anatomy, University of West Attica, Athens, GRC; 3 Vascular Unit, Third Department of Internal Medicine, General Hospital of Thoracic Diseases "Sotiria", National and Kapodistrian University of Athens, Athens, GRC; 4 Center of Diagnosis, Prevention and Treatment of Lymphedema-Lymphatic Diseases, Metropolitan Hospital, Athens, GRC; 5 Department of Nursing, National and Kapodistrian University of Athens, Athens, GRC; 6 Laboratory of Anatomy, Advanced Anatomical Applications, Artificial Intelligence and Experimental Surgical Research (AAAAAIES Lab), National and Kapodistrian University of Athens, Athens, GRC; 7 Department of Informatics, University of Piraeus, Piraeus, GRC; 8 Department of Intensive Care and Pulmonology, General Oncological Hospital of Kifisia "Oi Agioi Anargyroi", Athens, GRC; 9 Department of Anatomy, National and Kapodistrian University of Athens, Athens, GRC; 10 Department of Surgery, General Oncological Hospital of Kifisia "Oi Agioi Anargyroi", Athens, GRC

**Keywords:** anatomy, cancer rehabilitation, lymphedema, muscles, physiotherapy, quality of life

## Abstract

Lymphedema remains a prevalent yet often overlooked consequence of cancer, silently burdening patients with more than just visible swelling. Beneath the surface, it disrupts mobility, weakens musculature, and impairs sensory function. This study synthesizes current evidence on the morphometric, biomechanical, and sensory alterations associated with cancer-related lymphedema and examines how these impairments intersect with patient-reported quality of life outcomes. Through a comprehensive literature search in PubMed and Scopus, studies published from 2000 onwards were screened for inclusion. Eligible articles assessed patients with stage I-III lymphedema and reported on range of motion, muscle strength, sensory perception, body composition, and quality of life. Twenty-three studies met the criteria. Findings consistently point to reduced grip strength, restricted shoulder mobility, altered sensory feedback, and an unfavorable shift in body composition, namely, increased fat mass relative to muscle in affected limbs. These physical disruptions were closely tied to declines in multiple quality of life domains, including emotional well-being, social participation, body image, and role functioning. Altogether, the evidence reveals that cancer-related lymphedema is a multidimensional condition with persistent functional and sensory repercussions. Early multidisciplinary interventions, with physiotherapy as a core component, appear essential not only for managing symptoms but also for preserving long-term physical autonomy and psychosocial resilience among survivors.

## Introduction and background

Lymphedema is a chronic condition characterized by the accumulation of lymphatic fluid in the interstitial spaces of tissues, primarily resulting from an imbalance between capillary filtration and the lymphatic system's drainage capacity [1]. It occurs in approximately 30.2% of patients who have undergone cancer treatment and has a profound impact on mobility, independence, and daily activities [2-4]. Clinically, lymphedema presents as swelling, pain, numbness, discomfort, and a sensation of heaviness or tightness in the affected limb, often accompanied by restricted range of motion and diminished sensory perception [5]. The presence of lymphedema contributes to significant morbidity, including activity limitations, social withdrawal, and financial burden [3,4]. These limitations negatively influence psychological health and overall well-being [6,7]. As cancer survival rates improve, the persistent effects of lymphedema on physical, emotional, and social functioning become increasingly important [8-11]. Systematic reviews have consistently reported reduced life satisfaction in patients, especially in areas related to body image and functional capacity [12]. Patients frequently report compromised social well-being, including negative body image and sexual self-perception, with psychological effects often manifesting as feelings of marginalization and social isolation [13]. Despite the recognized burden of these symptoms, limited research has explored their direct correlation with quality of life. Therefore, the aim of this study is to examine the effect of cancer-related lymphedema on the morphometric and biokinematic (biomechanical) characteristics of the affected limb and its relationship with patients' quality of life, as well as to explore how anatomical and functional alterations interact with patients' experience and perceived well-being.

Methods

Data Sources and Search Strategy

The present review aims to summarize and interpret findings from the literature in order to provide a comprehensive understanding of the field. An extensive literature search was performed for studies published from 2000 onwards using PubMed and Scopus databases. Search terms included combinations of the following keywords: upper extremity lymphedema, lower extremity lymphedema, range of motion, strength, handgrip strength, sensation, body composition, quality of life in patients with lymphedema, and health-related quality of life. A total of 5,473 records were identified (Scopus: 3,111; PubMed: 2,362). After removing duplicates, abstracts, and systematic reviews, 314 studies remained. Following title and abstract screening, 27 full-text articles were assessed for eligibility. Ultimately, 23 studies met the inclusion criteria and were included in this review. Exclusions were based on the absence of a confirmed lymphedema diagnosis (n=1), inclusion of patients with negative lymph nodes (n=1), or omission of complex decongestive therapy as an intervention (n=2).

Inclusion Criteria

Eligible studies included those that evaluated at least one of the following lymphedema-related outcome measures: range of motion, muscular strength, sensory perception, body composition, or quality of life. Studies were included if they involved patients with stage I-III lymphedema and were published in English from 2000 onwards. Both cross-sectional and longitudinal designs were considered.

Exclusion Criteria

Systematic reviews, meta-analyses, doctoral theses, and conference abstracts were excluded. Additionally, studies that did not specifically address lymphedema or that focused on cancer without lymphedema-specific outcomes were excluded.

## Review

Study characteristics

The 23 included studies focused on patients with upper limb lymphedema [14-30] and lower limb lymphedema [31-36] (Table 1).

**Table 1:** Overview of clinical studies on lymphedema diagnosis, morphometric analysis, and quality of life evaluation n: number of participants (sample size); BCRL: breast cancer-related lymphedema; BIPQ: Brief Illness Perception Questionnaire; BRE: Body Image after Breast Cancer Questionnaire; CA: cancer; CDP: complete decongestive physiotherapy; CDT: complete decongestive therapy; CESD: Center for Epidemiologic Studies Depression Scale; CT: computed tomography; CX: chemotherapy context; DASH: Disabilities of the Arm, Shoulder, and Hand; DASS-21: Depression Anxiety Stress Scale; DEX: dual-energy X-ray absorptiometry; EORTC QLQ-C30: European Organisation for Research and Treatment of Cancer-Quality of Life Questionnaire, Core 30; FACT: Functional Assessment of Cancer Therapy; FACT-B: Functional Assessment of Cancer Therapy-Breast; FET: forced expiratory time; FL: forearm length; GCLQ: Gynecologic Cancer Lymphedema Questionnaire; HRQ: health-related quality; HRQOL: health-related quality of life; ICF: International Classification of Functioning, Disability and Health; IES: Impact of Event Scale; II, III: clinical stage II, III; JAMAR: Jamar hand dynamometer; JT: joint test; JTHFT: Jebsen-Taylor Hand Function Test; LBCQ: Lymphedema Breast Cancer Questionnaire; LEFS: Lower Extremity Functional Scale; LL: lower limb; LYMPH: general reference to lymphatic system measurements; LYMQOL: Lymphedema Quality of Life Questionnaire; MH: mental health; MRI: magnetic resonance imaging; POMS-SF: Profile of Mood States-Short Form; PSQI: Pittsburgh Sleep Quality Index; QLQ: Quality of Life Questionnaire; QLQC: Quality of Life Questionnaire-Core; SF: Short Form (e.g., SF-36); SGSE: Self-Generated Self-Efficacy Scale; SH: shoulder height; SWM: Semmes-Weinstein Monofilament; TUG: Timed Up and Go; VAS: Visual Analog Scale; WCLS: Wesley Clinic Lymphedema Scale; WI: Wisconsin Index; Lymph-ICF-LL: Lymphoedema Functioning, Disability and Health Questionnaire for Lower Limb Lymphoedema; FACT-G: Functional Assessment of Cancer Therapy-General

Authors	Goal/planning	Specimen/age/leaf	Identification of lymphedema	Evaluation of biometric/morphometric characteristics/quality of life	Results
Ridner 2005 [27]	To compare quality of life and symptoms between breast cancer survivors who developed chronic lymphedema and received treatment, with those who did not develop lymphedema. Descriptive study	n=128 women; mean age: 57 years (n=64 with lymphedema; n=64 without lymphedema)	Bioelectric impedance device (ImpediMed, Mansfield, Australia)	FACT-B, WCLS, short-form skin/arm condition symptom checklist, CESD, POMS-SF	Women with lymphedema scored significantly lower on all quality of life instruments
						Smoot et al. 2010 [28]	To compare differences in upper extremity lesions between women who developed lymphedema after breast cancer treatment and those who did not develop lymphedema. Also, to determine the impact of these impairments on self-reported limitations and daily activities	n=144 women; average age: 56 years (n=73 with lymphedema; n=71 without lymphedema)	Circumference measurements, bioelectric impedance device (ImpediMed SFB7), DASH	Purdue Pegboard (North Coast Medical, Morgan Hill, California, United States), finger tapping test (Psychological Assessment Resources, Inc., Lutz, Florida, United States), MicroFET2 dynamometer, goniometer, SWM (North Coast Medical, Morgan Hill, California, United States)	Significant differences in strength, range of motion, and sensation were found between affected and unaffected upper limbs and between patients with and without lymphedema. In the non-lymphedema group, the affected side showed less shoulder abduction strength and lower shoulder flexion, abduction, and external rotation range of motion, as well as total range of motion score (p<0.05) compared to the unaffected side. For the lymphedema group, the affected side had less strength in elbow flexion, wrist flexion, and two of the three grip tests, resulting in an overall reduction in upper extremity strength compared to the unaffected side. There was less sensitivity in the SWM test on the medial side of the arm, forearm, and index finger in the lymphedema group. In addition, there was less range of motion of the affected shoulder (flexion, abduction, and external rotation), wrist, index, and proximal interphalangeal joint flexion as well as a lower total range of motion score (p<0.05)
												Lee et al. 2012 [24]	To determine the effect of lymphedema on HRQOL over one year after breast cancer surgery. Contemporary study	n=200 women; average age: 53 years (n=104 healthy; n=96 with breast cancer (n=58 with lymphedema; n=38 without lymphedema))	Circumference measurements	SF-36	Between patients with and without lymphedema, there were no statistically significant differences in all scales of the SF-36. There were differences only when comparing breast cancer survivors with the healthy population, in all scales of the SF-36 except the vitality and mental health
Rowlands et al. 2014 [34]	To evaluate and compare the quality of life of women with self-reported lower limb lymphedema and with women with lower limb swelling compared with women without lymphedema or lower limb swelling after treatment for endometrial cancer 3-5 years after diagnosis. Contemporary study	n=639 women; mean age: 65 years (n=394 without lymphedema or edema; n=177 edema only; n=68 with lymphedema)	Self-reference	SF-12	Women with lymphedema had clinically lower levels of overall physical quality of life (p<0.05) than women without lymphedema and also scored significantly lower on three of the eight subscales (physical functioning, physical role limitations, and social functioning) than women without lymphedema or lower extremity edema (p<0.05). Women with lymphedema scored lower on the physical functioning subscale and had significantly lower overall physical and mental quality of life and significantly lower scores on all eight subscales than women without. Mental quality of life did not differ significantly between groups						
Gomes et al. 2014 [21]	To investigate changes in body composition and grip strength in breast cancer patients with and without lymphedema compared to healthy controls. Contemporary study	n=95 women, aged 51-54 years (n=46 healthy; n=49 with breast cancer (n=10 with lymphedema; n=39 without lymphedema))	Circumference measurements	Hydraulic hand dynamometer SH5001, DEX	Compared between the two groups, handgrip strength was lower in the breast cancer group than in the control group (p=0.0001). Trunk lean mass was decreased in the breast cancer group compared to the control group (p=0.04). Total lean mass was influenced by the type of surgery and was higher in women who underwent right mastectomy compared to those with left (p=0.04). However, in women with lymphedema, total lean mass (p=0.004), trunk fat mass (p=0.05), trunk lean mass (p=0.005), right arm lean mass (p=0.03), and left arm lean mass (p=0.01) increased six months after breast cancer surgery, while handgrip strength remained unchanged regardless of the presence or absence of lymphedema						
												Kim et al. 2015 [33]	To evaluate the effect of lower extremity lymphedema on quality of life in survivors of gynecological cancer. Contemporary study	n=53 women, aged 28-80 years (n=25 with lymphedema of the lower extremities; n=28 without lymphedema)	Perometry, lymphoscintigraphy, MRI, CT	GCLQ, EORTC QLQ-C30	According to the GCLQ scale, the total symptom score was higher in the lymphedema group than in the control group. Among the seven scales, scores for general edema, limb swelling, and weight were significantly higher in the lymphedema group, while scores for physical function, infection, pain, and numbness did not differ significantly between the two groups. In the EORTC QLQ-C30, the scores of the five functioning scales, three symptom scales, and five symptoms were not statistically different between the two groups. However, a significant economic burden was observed in the lymphedema group than in the control group (mean: 16.0 vs. 6.0; p=0.035). In addition, global health status was poorer in the lymphedema group, with borderline statistical significance (mean: 62.7 vs. 71.4; p=0.069)
Bojinović-Rodić et al. 2016 [17]	To assess HRQOL in patients with BCRL and its association with upper extremity function and edema size. Contemporary study	n=54 patients with lymphedema; mean age 56 years (90.74% had mild or moderate lymphedema; 9.26% had severe lymphedema)	Circumference measurements	SF-36, QuickDASH	A higher HRQOL score was reported for mental health (47.0±12.2) than physical health (42.2±7.5) (p<0.05). The highest SF-36 values were found in the mental health (67.7±22.9) and social functioning (70.1±23.1) domains. The lowest scores were recorded in the physical role (46.9±39.1) and general health (49.3±20.1) domains												
						Borri et al. 2017 [18]	To propose an MRI acquisition and analysis protocol using image segmentation that measures and visualizes fluid, fat, and muscle volumes in the limb with lymphedema and to compare affected and unaffected arms with lymphedema, providing an analysis of both volume and the distribution of the different tissue components	n=13 women with lymphedema, aged 34-77 years	MRI	MRI	Measurements showed that most of the swelling was within the fascial volume. The fascial volume showed an increase (overall, 94% of the excess volume) that constituted the gross edema, and fat was the major component of the edema in the total fascial volume. Total volume was significantly different (p<0.0005) between affected and unaffected limbs, with greater volume in affected limbs. Muscle volume was not significantly different between the two arms. In the results, it was found that lymphedema does not uniformly affect the arm and the fluid mainly collects around the area of the forearm and elbow, while the fat mainly surrounds the upper part of the arm						
Popović-Petrović et al. 2018 [26]	To identify the differences in quality of life in women with BCRL and in women without lymphedema and to determine the contribution of psychological support. Contemporary study	n=64 women; average age: 60 years (n=34 with lymphedema; n=30 without lymphedema)	Circumference measurements	FACT-B, BIPQ, DASS-21, SGSE	There was no statistically significant difference both in the overall quality of life of women with lymphedema and those without lymphedema and in individual subscales (t-test, Mann-Whitney U test (p>0.05)) with illness perception and depression being important predictors of quality of life												
						Pedrosa et al. 2019 [31]	To evaluate the impact of unilateral lower extremity lymphedema on functionality and quality of life. Descriptive study	n=25 patients; mean age: 52 years (n=18 women; n=7 men)	Circumference measurements	SF-36, Lymph-ICF-LL, TUG test	Physical role (25.0±31.4), emotional role (36.0±42.9), and functional ability (45.4±25.9) were most affected according to SF-36. Mobility (6.0±2.6) and mental health (5.6±2.5) were more affected than life domains/social life (3.9±2.4) which were the least affected in Lymph-ICF-LL. The mean TUG time was 9.88±1.98 s, which is considered satisfactory
												Giray and Akyüz 2019 [20]	To assess the relationships between caregiver burden, quality of life, arm disability, grip strength, and lymphedema symptoms in women with lymphedema after mastectomy. Cross-sectional study	n=52 women with lymphedema who were divided into three groups according to lymphedema stage (0-I, II, II); mean age: 48 years	Circumference measurements	DASH, EORTC QLQ-C30 and EORTC QLQ BRE-23, VAS, JAMAR	Patients in stages 0-I, II, and III were found to be similar in grip strength of the affected and unaffected side. Stage III patients showed lower EORTC QOL-C30 scores, indicating a lower quality of life in patients with lymphedema. Patients in stage III had poorer functioning and greater disability compared to those in stages I and II. Stage III patients experience greater disability and worse health-related quality of life, and lymphedema symptoms were found to be highly correlated with lymphedema patients' quality of life
Baklaci et al. 2020 [14]	To show whether CDT contributes to volume and handgrip strength change in patients with lymphedema	n=74 patients with lymphedema; mean age: 56 years	Circumference measurements	JAMAR	The unaffected arm was stronger than the arm with lymphedema (p<0.01). Patients had lower grip strength in the limb with lymphedema than in the healthy limb, and this difference persisted after treatment												
Watson et al. 2019 [36]	Primary objective was to estimate the incidence of lower extremity lymphedema after endometrial cancer surgery and secondary objectives were to analyze postoperative quality of life and lower extremity function. Longitudinal pilot study	n=97 women; mean age: 62 years	Circumference measurements	LEFS, FACT-G	LEFS scores significantly decreased by an average of 9.1 points from baseline to the 4-6-week measurement period (95% CI: 5.6-12.7). This trend was not maintained at later measurement periods, and no significant differences in quality of life were observed at any of the measurement periods. The presence of lymphedema was significantly associated with worse lower extremity function at 4-6 weeks and 6-9 months, with a mean reduction of 27% at 4-6 weeks and 13% reduction at 6-9 months, compared with the median LEFS scores 4% and 0% in patients without lymphedema at these measurement periods. This change caused difficulty in performing activities of daily living and deterioration of general mobility. This difference did not persist at 12-18 months. No correlation was found between lymphedema and global quality of life
Mistry et al. 2021 [25]	To assess the grip strength and functionality in patients with lymphedema. Cross-sectional study	n=62 women; mean age: 55 years (n=31 with lymphedema; n=31 without lymphedema)	Not mentioned	Hand dynamometer test, JTHFT	There were significant decreases in both strength and precision grip strength (p<0.05) in the lymphedema patients compared to healthy controls. Hand functions were significantly reduced in all activities in women with lymphedema compared to healthy women (p<0.05)
						Baran et al. 2021 [15]	To compare the three-dimensional scapular kinematics after breast cancer surgery in moderate and severe lymphedema groups and in patients without lymphedema. Secondary objectives were to compare shoulder range of motion and upper extremity function between groups as well as correlations between scapular kinematics and upper extremity function. Contemporary study	n=67 women, aged 42-68 years (n=22 without lymphedema; n=18 moderate lymphedema; n=27 severe lymphedema)	Water displacement	Baseline digital inclinometer (Ascension Technology Corporation, Burlington, Vermont, United States), QuickDASH	The no or moderate lymphedema group had significantly higher range of motion in active shoulder abduction than the severe lymphedema group (p=0.005 and p=0.007). The affected shoulder of the patients had lower active range of motion values than the values of the unaffected shoulder for all measurements (p<0.05)
												Baran et al. 2021 [16]	To investigate whether and how the presence of lymphedema affects upper extremity sensation and to assess whether CDP has a favorable impact on sensory testing	n=27 women with stage II lymphedema; mean age: 59 years	Circumference measurements	Ultrasound with 5-12 MHz linear probe (Logiq P5, GE Medical Systems, Milwaukee, Wisconsin, United States), SWM, esthesiometer algometer (JTech Algometer Commander, JTech Medical, Midvale, Utah, United States) tactile localization test with pencil and ruler	Before treatment, the volume and thickness of the skin, epidermis, and subcutaneous fat were found to have higher values (p<0.05) in the affected limbs compared to those in the unaffected limbs. The affected sides showed significantly higher values for SWM (p<0.001), static two-point discrimination (p=0.002), moving two-point discrimination (p=0.011), pressure pain threshold (p=0.001), and touch detection (p<0.001). These findings indicate that sensory perception of the upper limb on the affected side is reduced in women with lymphedema
																		Jørgensen et al. 2021 [23]	To investigate the effect of lymphedema on HRQOL up to 10 years after breast cancer treatment	n=1067 women; mean age: 65 years (n=244 with lymphedema; n=823 without lymphedema)	Circumference measurements	LYMPH-ICF, DASH, SF-36	Patients with lymphedema reported worse physical functioning, general mental health, social role functioning, reduced recreational, social and household activities, reduced physical role, general health perception, reduced occupational activity, and increased physical pain compared to patients without lymphedema. Increased severity of lymphedema was associated with more severe symptoms
												Tamam et al. 2021 [29]	To assess the quality of life and sleep quality in Saudi Arabian women at different stages of lymphedema after breast cancer treatment. Contemporary study	n=163 women with stage I-III lymphedema; mean age: 42 years	Not mentioned	EORTC QLQ-C30, PSQI 19	Patients with stage I lymphedema (n=27) reported the best scores in functional status, physical status, role-related, emotional, cognitive, and social scales (64.2±9.4, 66.3±10.2, 71.6±11.4, 68.5±10.8, and 65.4±9.8, respectively). They also showed more favorable results in symptom scales, including loss of appetite, fatigue, dyspnea, nausea/vomiting, pain, insomnia, and constipation (74.5±10.6, 70.6±9.7, 73.2±10.4, 68.4±9.5, 71.5±9.8, 74.7±10.2, and 45.3±7.5, respectively). The overall quality of life score was 53.7±7.4. Patients with stage II lymphedema (n=84) showed lower mean values compared to those with stage I on the functional and physical status and role-related scales. Stage III lymphedema patients (n=52) showed the lowest quality of life scores compared to patients with stage I and stage II lymphedema across all scales (p<0.5)						
Anbari et al. 2021 [30]	To examine women with newly diagnosed BCRL regarding their quality of life over seven years. Qualitative longitudinal study	n=97 women with lymphedema; mean age: 53 years	Circumference measurements, perometry	LBCQ	Initially, lymphedema had an impact on physical function, including pain, fatigue, and reduced functionality. Second, lymphedema has an impact on daily life and social functioning (participants feel and are restricted in their jobs and roles while also expressing concerns about body image). Finally, it has an impact on the psychology of patients with feelings of frustration and depression, and they are more irritable																		
Togawa et al. 2021 [22]	To investigate which symptoms of lymphedema affect the quality of life of patients with lymphedema. Contemporary study	n=499 women, aged 35-64 years (n=362 without lymphedema; n=137 with lymphedema (n=40 without symptoms of lymphedema; n=97 with at least one symptom of lymphedema))	Self-reference	SF-36, Perceived Stress Scale, Fear of Recurrence Scale, Cancer Rehabilitation Evaluation System's Sexual Functioning Summary Scale, lymphedema‑specific HRQOL	Women reporting lymphedema with or without symptoms had worse scores on the functioning, physical role, physical pain, general health, and social functioning subscales (all p≤0.01) of the SF-36 than women without lymphedema (all p<0.05). In the physical functioning and general health scales of the SF-36, there were differences only between women without lymphedema and those who reported at least one symptom of lymphedema (p≤0.03). All symptoms except dry skin were associated with poorer quality of life (all p≤0.0002). Lymphedema-specific quality of life was lower in women with at least one lymphedema symptom than in women without lymphedema symptoms (p<0.0001)																		
						Carter et al. 2021 [32]	To assess the quality of life in patients who developed lower extremity lymphedema after gynecological cancer surgery. Cohort study	n=768 women, aged 30-80 (n=338 with lymphedema; n= 430 without lymphedema)	Circumference measurements	FACT-G, GCLQ, IES, Body Image subscale, Sexual and Vaginal subscale, LEFS	Women living with symptoms of lower extremity lymphedema reported lower health-related quality of life (p<001), higher cancer distress (IES) (p<0.001), and more lower extremity dysfunction (LEFS) (p<0.001), highlighting how this condition affects their daily life. The results showed that women with symptoms of lower extremity lymphedema had poorer body image (p<0.001) and worse sexual health (p<0.001) compared to women without lymphedema												
												Crescenzi et al. 2022 [19]	To assess the subcutaneous adipose tissue in women with lymphedema	n=46 women, average age: 50 years (n=22 women with stage I-II lymphedema; n=24 healthy women)	Bioimpedance spectroscopy, perometry (L-Dex)	MRI	Muscle tissue was similar between groups on average, but the fat-to-muscle fraction was significantly increased on the affected side of participants with lymphedema relative to healthy participants. It was observed that the fat-to-muscle fraction was increased on the affected side relative to the contralateral side of participants with increasing severity of lymphedema. Also, areas of nodular adipose tissue with fluid and fibrosis appear more often in advanced stages of lymphedema
																		Sponholtz et al. 2022 [35]	To assess the patient-reported incidence and severity of early lymphedema and its impact on quality of life in women undergoing surgery for early-stage cervical cancer. Cohort study	n=200 women; mean age: 43 years (n=36 with lymphedema; n=164 without lymphedema)	Self-reference	LYMQOL, EORTC QLQ-C30 and QLQ-CX24	According to LYMQOL, women with severe lymphedema had reduced physical functioning (p=0.001) and appearance (p=0.007). The impact of lymphedema on quality of life as assessed by the EORTC QLQ-C30 and QLQ-CX24 questionnaires showed that early lymphedema was associated with significant impairment in body image (p=0.002), global health status (p=0.04), physical functioning (p=0.008), role (p=0.04), cognitive (p=0.04), and social functioning (p=0.007), as well as a higher level of fatigue (p=0.01), pain (p=0.04), dyspnea (p=0.03), and symptom experience (p=0.007) between women with lymphedema and those without. 18% of women developed early lymphedema, negatively affecting several aspects of their quality of life physically, psychosocially, and sexually

Participants were all adults (>18 years), with an age range of 52-65 years. Sample sizes varied from 13 to 1,067 participants, with most being female. Lymphedema diagnosis varied across studies. Thirteen studies utilized peripheral measurements [14,16,17,19-22,24,26,28,30,32,36], two relied on self-reported diagnosis [23,35], and others used water displacement [15], bioelectrical impedance analysis [27], or bioimpedance spectroscopy (L-Dex) [19,28]. Magnetic resonance imaging (MRI) was used alone or in combination with computed tomography (CT), perimetry, and lymphoscintigraphy in two studies [33,34]. Two studies did not specify the diagnostic method [25,29].

A total of 4,530 participants were assessed, of whom 1,242 had upper limb lymphedema and 512 had lower limb lymphedema. Five studies evaluated grip strength using a dynamometer [14,20,21,25,28]. Three assessed body composition via dual-energy X-ray absorptiometry (DEXA), MRI, or both [18,19,21]. Shoulder range of motion was evaluated in two studies using a goniometer [24,28], and sensation was assessed in two studies using Semmes-Weinstein monofilaments [16,28]. Nine studies evaluated quality of life in patients with upper limb lymphedema [17,20,22-24,26,27,29,30], and six in patients with lower limb lymphedema [31-36].

Strength

Evidence on the impact of lymphedema on muscular strength is mixed. One study found that healthy limbs exhibited significantly greater strength than those with lymphedema (p<0.01) [14]. Mistry et al. reported significant reductions in both grip and precision grip strength in women with lymphedema compared to healthy controls (p<0.05) [25]. Smoot et al. observed reduced strength in elbow and wrist flexion on the affected limb (p<0.05) [28]. In contrast, Giray and Akyüz reported no significant differences in grip strength across all stages of lymphedema [20], and Gomes et al. found no variation in grip strength with or without lymphedema [21].

Range of motion

Range of motion was examined in two studies [15,28]. Baran et al. found that shoulders on the affected side had significantly reduced active range of motion in flexion, abduction, and rotation (p<0.05), with more severe lymphedema correlating with greater impairment (p=0.007) [15]. Smoot et al. confirmed these findings, reporting significantly diminished mobility in the shoulder, wrist, index finger, and proximal interphalangeal joints of the affected limbs, with the most pronounced limitations seen in shoulder abduction [28].

Body composition

Three studies examined changes in body composition [20,22,32]. One study reported reduced trunk muscle mass in patients with lymphedema compared to controls (p=0.04), although the relationship between body composition changes and lymphedema status remained inconclusive [22]. Another found that total fascial volume (fluid and fat) was significantly increased in the affected limbs, with adipose tissue being the predominant component of the edema; no significant difference in muscle volume was detected [32]. Crescenzi et al. demonstrated a significantly higher fat-to-muscle ratio in the lymphedema-affected limb (0.732±0.184) compared to the unaffected limb (0.639±0.167; p=0.007). In contrast, healthy controls exhibited symmetrical values between limbs (p=0.51) [19].

Sensibility

Sensory perception of the affected upper limb appears to be impaired in women with lymphedema, highlighting the need for further investigation into its impact on daily life. One study demonstrated that the affected limbs exhibited significantly higher values in Semmes-Weinstein Monofilament (SWM) testing (p<0.001), static two-point discrimination (p=0.002), moving two-point discrimination (p=0.011), pressure pain threshold (PPT) (p=0.001), and touch localization (p<0.001) [16]. Similarly, Smoot et al. reported decreased sensitivity in the Semmes-Weinstein test on the medial side of the arm, forearm, and index finger in the lymphedema group [28].

Quality of life

Out of nine studies evaluating the quality of life in patients with upper extremity lymphedema, five compared quality of life between patients with and without lymphedema [22-24,26,27], while two investigated quality of life across different lymphedema stages [20,29]. Although results vary, the majority of these studies indicate that patients with lymphedema experience significantly worse quality of life, including reduced physical functioning and compromised psychological and social well-being compared to those without lymphedema [22,23,27]. One study found that women with lymphedema had poorer quality of life scores across all measured domains compared to those without lymphedema [27]. Another study demonstrated that lymphedema is associated with long-term impairment in quality of life, particularly within physical and psychosocial domains, with the General Health Perception Scale scores being significantly worse even among patients reporting at least one symptom of lymphedema (p<0.0001) [22]. Togawa et al. reported that women with lymphedema, regardless of symptom presence, exhibited lower Short Form (36) Health Survey (SF-36) scores, specifically in functioning, physical role, pain, general health, and social functioning domains (all p≤0.01), compared to women without lymphedema (all p<0.05) [22]. Conversely, Popović-Petrović et al. found no statistically significant difference in overall quality of life scores between groups (t=0.469; p>0.05), although illness perception (β=-0.603; t=-5.958; p<0.001) and depression (β=-0.411; t=-4.101; p<0.001) significantly contributed to quality of life variance [26]. Similarly, Lee et al. reported no significant differences across all SF-36 scales [24]. Anbari et al. revealed that lymphedema negatively impacts physical function, daily activities, social interactions, and psychological status, manifesting as frustration and depression [30]. Another study observed statistically higher quality of life scores in the mental health domain (47.0±12.2) compared to the physical domain (42.2±7.5) (p<0.05). The highest SF-36 scores were recorded in mental health (67.7±22.9) and social functioning (70.1±23.1), whereas role limitations, physical functioning, and general health domains demonstrated lower scores (46.9±39.1 and 49.3±20.1, respectively) [17].

Two studies assessed quality of life across different lymphedema stages [20,29]. Giray and Akyüz reported that patients with stage III lymphedema exhibited greater disability and poorer quality of life compared to stages I and II, with significantly lower functional scale scores on the European Organisation for Research and Treatment of Cancer-Quality of Life Questionnaire, Core 30 (EORTC QLQ-C30) (36.88±32.07 vs. 66.91±22.01 and 66.46±15.85 for stages I and II, respectively) [20]. Similarly, Tamam et al. identified statistically significant differences between stages I, II, and III (p<0.05), with stage III patients demonstrating lower scores in functional and physical status, role-related, emotional, cognitive, and social functioning (57.3±8.5, 59.6±8.7, 65.2±9.4, 61.6±8.3, and 58.5±7.9), as well as symptom and global health scales (32.4±5.6) compared to stages I and II [29].

Regarding lower extremity lymphedema after genital cancer treatment, six studies evaluated quality of life outcomes [17,32-36]. Pedrosa et al. identified the most affected SF-36 domains as physical role (25.0±31.4), emotional role (36.0±42.9), and functional ability (45.4±25.9), while the Lymphoedema Functioning, Disability and Health Questionnaire for Lower Limb Lymphoedema (Lymph-ICF-LL) indicated greater impairment in mobility (6.0±2.6) and mental function (5.6±2.5), with lesser impact on life activities and social life (3.9±2.4) [31].

Carter et al. reported that women experiencing lower extremity lymphedema symptoms had lower health-related quality of life (p<0.001), increased cancer-related distress (IES) (p<0.001), greater lower extremity dysfunction (LEFS) (p<0.001), poorer body image (p<0.001), and diminished sexual health (p<0.001) compared to women without lymphedema [32].

Kim et al. found that patients with lower extremity lymphedema reported more symptoms according to the Korean version of the Gynecologic Cancer Lymphedema Questionnaire (GCLQ-K) total score (mean: 5.32 vs. 1.86; p<0.001). Although global health status on the EORTC QLQ-C30 was poorer in the lymphedema group (mean: 62.7 vs. 71.4; p=0.069), no significant differences were observed in five functioning scales (physical, role, emotional, cognitive, social), three symptom scales (fatigue, pain, nausea/vomiting), or five individual symptoms (dyspnea, insomnia, loss of appetite, constipation, diarrhea) [33]. Additionally, a greater economic burden was noted in the lymphedema group (mean: 16.0 vs. 6.0; p=0.035) [33].

Another study demonstrated that women with lower extremity lymphedema had lower overall physical quality of life (M=41.8; SE=1.4) and reduced scores on three of the eight SF-12 (Short Form (12) Health Survey) subscales (physical functioning, physical role, social functioning) compared to women without lymphedema or edema (M=45.1; SE=0.8; p=0.07), while mental quality of life remained within normative ranges (M=49.6; SE=1.1; p=1.0) [34].

Sponholtz et al. found that early-stage lymphedema was associated with significant impairments in body image (p=0.002), global health status (p=0.04), physical condition (p=0.008), physical role (p=0.04), cognitive (p=0.04), and social functioning (p=0.007), along with higher levels of fatigue (p=0.01), pain (p=0.04), dyspnea (p=0.03), and overall symptom burden (p=0.007) [35]. Lastly, Watson et al. reported that the presence of lymphedema was significantly associated with worse lower extremity function at 4-6 weeks and 6-9 months postoperatively; however, global quality of life as assessed by the Functional Assessment of Cancer Therapy-General (FACT-G) did not differ significantly at either time point [36].

Discussion

This review highlights the far-reaching impact of lymphedema on physical function and patient well-being following cancer treatment. Across the included studies, consistent patterns of morphometric and biokinematic deterioration emerged, including decreased grip strength, impaired joint mobility, especially in the shoulder, and reduced sensory discrimination [14-16,22,26-28]. These deficits suggest not only structural tissue changes but also neuromuscular maladaptation.

A particularly salient finding was the increase in subcutaneous adipose tissue relative to lean mass in lymphedematous limbs, possibly reflecting chronic inflammation, fibrosis, or physical disuse [14-16,22,28]. While muscle volume was not universally reduced, its functional output appeared compromised, suggesting that qualitative muscle deterioration (e.g., fatty infiltration or denervation) may play a role. Notably, these physical impairments were closely linked to declines in quality of life. Domains such as physical functioning, emotional resilience, and social participation were especially vulnerable. In advanced lymphedema stages, these effects were magnified, underlining the progressive nature of the condition [22,31,35].

However, the data also reveal significant heterogeneity. Variability in assessment tools, sample sizes, diagnostic criteria, and follow-up duration limits the ability to generalize findings. Most notably, the absence of pre-treatment baselines and the reliance on the contralateral limb as a control group introduce potential bias, particularly given the documented bilateral effects of surgery or radiation [28]. Importantly, few studies utilized lymphedema-specific quality of life instruments; only three employed the Lymphedema Quality of Life Questionnaire, while most relied on general health measures, limiting sensitivity to lymphedema-specific symptoms [22,31,35]. Moreover, only one study addressed the economic burden of the condition, a critical oversight given its chronic nature and resource-intensive management [22].

From a clinical perspective, this synthesis underscores the necessity of a proactive, multidisciplinary approach: incorporating physical therapy, exercise, and weight management to maintain strength and mobility [14-16,22,26,27], alongside psychological support to address emotional burden and body image concerns.

Future studies should adopt standardized diagnostic and assessment protocols, include longitudinal designs with pre-treatment baselines, and apply lymphedema-specific quality of life tools. Investigating mechanistic underpinnings, such as the role of inflammation, neuromuscular dysfunction, or metabolic alterations, would enhance our understanding and support more targeted interventions.

## Conclusions

Based on the findings of this review, it is evident that lymphedema constitutes a significant and debilitating complication among patients' post-cancer treatment. The condition is associated with measurable reductions in range of motion, muscular strength, and sensory perception, alongside notable alterations in body composition. These functional and morphological changes collectively contribute to a marked decline in patients' quality of life. Furthermore, volumetric changes in the affected limb profoundly impact body image, which, together with deteriorations in mental health and social functioning, represent the domains most adversely affected according to the majority of studies reviewed. These findings underscore the critical importance of implementing comprehensive and multidisciplinary management strategies, incorporating physical therapy, weight management, and psychosocial support, to effectively mitigate the impact of lymphedema and enhance patient outcomes.
